# Distribution and Relative Abundance of S100 Proteins in the Brain of the APP23 Alzheimer’s Disease Model Mice

**DOI:** 10.3389/fnins.2019.00640

**Published:** 2019-06-20

**Authors:** Simone Hagmeyer, Mariana A. Romão, Joana S. Cristóvão, Antonietta Vilella, Michele Zoli, Cláudio M. Gomes, Andreas M. Grabrucker

**Affiliations:** ^1^Cellular Neurobiology and Neuro-Nanotechnology Lab, Department of Biological Sciences, University of Limerick, Limerick, Ireland; ^2^WG Molecular Analysis of Synaptopathies, Department of Neurology, Neurocenter of Ulm University, Ulm, Germany; ^3^Biosystems and Integrative Sciences Institute, Faculdade de Ciências, Departamento de Química e Bioquímica, Universidade de Lisboa, Lisbon, Portugal; ^4^Department of Biomedical, Metabolic and Neural Sciences, Center for Neuroscience and Neurotechnology, University of Modena and Reggio Emilia, Modena, Italy; ^5^Bernal Institute, University of Limerick, Limerick, Ireland; ^6^Health Research Institute (HRI), University of Limerick, Limerick, Ireland

**Keywords:** S100A8, S100A6, S100B, amyloid beta, cerebellum, aggregates, zinc

## Abstract

Increasing evidence links proteins of the S100 family to the pathogenesis of Alzheimer’s disease (AD). S100 proteins are EF-hand calcium-binding proteins with intra- and extracellular functions related to regulation of proliferation, differentiation, apoptosis, and trace metal homeostasis, and are important modulators of inflammatory responses. For example, S100A6, S100A8, and S100B expression levels were found increased in inflammatory diseases, but also neurodegenerative disorders, and S100A8/A9 complexes may provide a mechanistic link between amyloid-beta (Aβ) plaque formation and neuroinflammation. On the other hand, S100B, a proinflammatory protein that is chronically up-regulated in AD and whose elevation precedes plaque formation, was recently shown to suppress Aβ aggregation. Here, we report expression of S100A6 and S100B in astrocytes and less so in neurons, and low level of expression of S100A8 in both neurons and glial cells *in vitro*. *In vivo*, S100A8 expression is almost absent in the brain of aged wildtype mice, while S100A6 and S100B are expressed in all brain regions and most prominently in the cortex and cerebellum. S100B seems to be enriched in Purkinje cells of the cerebellum. In contrast, in the brain of APP23 mice, a mouse model for Alzheimer’s disease, S100B, S100A6, and S100A8 show co-localization with Aβ plaques, compatible with astrocyte activation, and the expression level of S100A8 is increased in neural cells. While S100A6 and S100B are enriched in the periphery of plaques where less fibrillar Aβ is found, S100A8 is more intense within the center of the inclusion. *In vitro* assays show that, similarly to S100B, S100A6, and S100A8 also delay Aβ aggregation suggesting a regulatory action over protein aggregation. We posit that elevated expression levels and overlapping spatial distribution of brain S100 proteins and plaques translates functional relationships between these inflammatory mediators and AD pathophysiology processes that uncover important molecular mechanisms linking the aggregation and neuroinflammation cascades.

## Introduction

Alzheimer’s disease (AD) is a common form of dementia, resulting in a progressive decline of cognitive functions. The pathological hallmarks include the presence of extracellular senile plaques and intracellular neurofibrillary tangles. Senile plaques are formed by aggregates of the amyloid-β (Aβ) peptide that result from cleavage of the Amyloid Precursor Protein (APP) through beta and gamma secretases, and a pathological cascade of aggregation steps of monomeric Aβ forming oligomers and finally fibrils (Masters et al., 2015). An increased neuroinflammatory response has been observed in AD, mediated by microglia and reactive astrocytes in the vicinity of senile plaques. There, Aβ induces the chronic expression and secretion of cytokines and chemokines – the so called alarmins or danger associated molecular patterns (DAMPS) – enhancing Aβ generation and possibly loss of synapses and neurons (Venegas and Heneka, 2017). Among these alarmins are S100, which are small EF-hand Ca^2+^-binding proteins whose multiple functions are tied to expression levels and intra- or extracellular localization (Heizmann et al., 2002; Donato et al., 2009, 2013).

Understanding the physiology of S100 proteins in the context of their concentration – dependent activities and the investigation of the transition between their trophic roles (at nanomolar intracellular levels) and deleterious pro-inflammatory activities (at micromolar extracellular levels) is particularly challenging in the context of AD pathophysiology, as it can open new therapeutic opportunities [recently reviewed by Cristóvão and Gomes (2019)]. S100B, S100A8, and S100A6 are among the most prominent brain expressed S100 proteins, which are all upregulated by aging and neuronal damage. These proteins also share the ability to bind Zn^2+^/Cu^2+^ in sites secondary to those of Ca^2+^-binding in the EF-hand motifs. This is an interesting characteristic considering that dyshomeostasis of neurometals is a well-established feature across neurodegenerative disorders and in particular in AD (Leal et al., 2012; Barnham and Bush, 2014; Cristovao et al., 2016).

S100B is one of the most abundant proteins in the brain (0.5%) and is constitutively produced by astrocytes at low levels. Brain injury and neurodegeneration result in astrocyte activation and increased expression of S100B, with its extracellular release and engagement of RAGE-mediated signaling and microglial activation (Leclerc et al., 2010). Several evidences implicate S100B in AD pathogenesis: it is systematically elevated in AD patients and animal models (Sheng et al., 1994; Van Eldik and Griffin, 1994; Peskind et al., 2001), it is present in elevated amounts in astrocytes surrounding neuritic plaques preceding their appearance (Sheng et al., 2000) and is suggested to regulate plaque formation. Knockout of S100B in the PS/APP AD mouse model selectively decreases plaque load in the cortical region (Roltsch et al., 2010) and its overexpression increases Aβ levels and deposits at early stages (Mori et al., 2010). While elevated levels of extracellular S100B trigger AD aggravating roles as a pro-inflammatory enhancer, novel protective functions for S100B at lower levels and early disease stages are emerging. Recently we have established that S100B is a regulator of elevated zinc levels in the brain and that this metal-buffering activity is tied to a neuroprotective role, through an indirect effect on calcium levels and in inhibition of excitotoxicity (Hagmeyer et al., 2017). Also, we have recently reported a calcium-tuned interaction between S100B and Aβ42 monomers, oligomers, and fibrils that suppresses Aβ42 aggregation and mitigates its cellular toxicity (Cristóvão et al., 2018). S100A6 is also upregulated in AD patients as well as in AD model mice (Boom et al., 2004; Wirths et al., 2010; Weissmann et al., 2016). The protein is found in clusters in astrocyte-positive regions within senile plaques. Its high affinity zinc binding properties led to the suggestion that it may play a role in the regulation of the homeostasis of this metal ion in AD (Boom et al., 2004). Interestingly, a zinc sequestering effect similar to that reported for S100B was recently elucidated: exogenous S100A6 protected cultured cells against Zn^2+^ toxicity and in APP/PS1 transgenic mice, increased S100A6 levels correlated with disaggregation of Aβ and decrease in plaque load (Zhi-Ying et al., 2019). Finally, S100A8 is also implicated in AD and its levels correlate with those of Aβ, being elevated in the hippocampus of Tg2576 and TgAPParc AD model mice (Lodeiro et al., 2017). Reciprocally, exposure of SH-SY5Y neuroblastoma cells to recombinant S100A8 increased Aβ42 and decreased Aβ40 production (Lodeiro et al., 2017). Studies in Tg2576 and TgAPParc AD mice brains indicate the presence of S100A8 inclusions distinct from corpora amylacea, that are formed independently of Aβ plaques (Lodeiro et al., 2017), a feature which is likely tied to the self-assembly propensity of the S100 family members (Fritz et al., 2010; Carvalho et al., 2013a, 2013b).

## Materials and Methods

### Materials

Primary antibodies were purchased from Sigma Aldrich (Map2, S100B), Novus Biologicals [S100A8 (calgranulin A)], Abcam (GFAP, S100B, S100A6), and Covance (β-III Tubulin). Secondary antibodies Alexa488 and Alexa568 were purchased from Life Technologies. Secondary HRP antibodies were purchased from DAKO. Unless otherwise indicated, all other chemicals were obtained from Sigma-Aldrich.

### Protein Expression and Purification

Recombinant Aβ42 was expressed in *Escherichia coli* and purified as described previously (Botelho et al., 2012). The human Aβ42 expression plasmid was a gift from J. Presto (Karolinska Institute, Sweden). To obtain the monomeric form, 1 mg of Aβ42 was dissolved in 7 M guanidine hydrochloride and eluted in a Superdex S75 (GE Healthcare) with 50 mM Hepes (pH 7.4). Low-bind tubes (Axygen Scientific, Corning) were used in all procedures employing Aβ42. Human S100B, S100A6, and S100A8 were expressed in *E. coli* BL21(DE3) and purified to homogeneity using previously established protocols and quantitated using reported extinction coefficients (Botelho et al., 2012; Brophy et al., 2012). Apo S100B, S100A6, and S100A8 were prepared by incubation at 37°C for 2 h with a 300-fold excess of dithiothreitol (DTT) and 0.5 mM EDTA and eluted in a Superdex S75 (GE Healthcare). To remove contaminant trace metals all solutions were passed through Chelex resin (Bio-Rad). S100 protein solutions were prepared and stored in 50 mM Tris–HCl pH 7.4.

### Aβ42 Aggregation Kinetics

Aβ42 aggregation kinetics was investigated by monitoring the fluorescence increase of the amyloid-sensitive dye Thioflavin-T (ThT) (Gade Malmos et al., 2017; Cristóvão et al., 2019) as a function of time in a plate reader (FLUOstar OPTIMA, BMG Labtech) using a 440 nm excitation filter and a 480 nm emission filter. Fluorescence was recorded using bottom optics in 96-well polyethylene glycol-coated black polystyrene plates with a clear bottom (Corning, 3881). Briefly, Aβ42 monomer was isolated by gel filtration (Tricorn Superdex75 column, GE Healthcare) in 50 mM Hepes (pH 7.4) immediately prior to experiments and diluted in the same buffer with 1.1 mM CaCl2. ThT (10 μM) was added to each condition. Monomeric Aβ42 (5 μM) was used in aggregation assays at 37°C, without agitation with fluorescence read every 400 s. For testing the effects of S100A6 and S100A8, the proteins were added to the reaction media at 15 μM each. S100B, whose effects on Aβ42 aggregation had been previously reported (Cristóvão et al., 2018), was tested under identical assay conditions as a comparison and control. Appropriate controls in the absence of Aβ42 did not reveal significant variations on ThT intensity due to S100 proteins alone. Triplicates were routinely performed for all assays. Data analysis was carried out using the AmyloFit platform, which implements the master equations derived from basin-hopping algorithm that describe the evolution of total fibril mass in the presence of primary and secondary nucleation events, and from which microscopic processes and reaction rates can be determined from global fitting (Meisl et al., 2016). The kinetic traces were fitted using the secondary nucleation model. The normalized intensity curves and corresponding fits were extracted from the platform and presented as normalized intensities representing fibrillar mass fraction, from which the reaction half times (*t*_1/2_) were estimated (Arosio et al., 2015).

### Hippocampal Culture From Rat Brain

Pregnant rats were purchased from Janvier Labs. The preparation of hippocampal cultures was performed as described before (Grabrucker et al., 2009) from rat (embryonic day-18; E18). After preparation the hippocampal neurons were seeded on poly-l-lysine (0.1 mg/ml; Sigma-Aldrich) glass coverslips in a 24 well plate at a density of 3 × 10^4^ cells/well. Cells were grown in Neurobasal^TM^ medium (Life Technologies), complemented with B27 supplement (Life Technologies), 0.5 mM L-Glutamine (Life Technologies) and 100 U/ml penicillin/streptomycin (Life Technologies) and maintained at 37°C in 5% CO_2_.

### Immunocytochemistry

Cells were fixed with 4% paraformaldehyde (PFA)/4% sucrose/PBS at 4°C for 20 min. After washing 2× 5 min with 1× PBS with 0.2% Triton X-100 at RT, blocking was performed with 10% FBS in 1× PBS at RT for 1 h, followed by the primary antibody for 2 h at RT. After a 3× 5 min washing-step with 1× PBS, incubation with the secondary Alexa488 and/or Alexa568 antibody followed for 1 h at RT. The cells were washed again in 1× PBS for 10 min and cell nuclei stained with DAPI for 5 min. After washing with aqua bidest., coverslips were mounted using VectaMount (Vector Labs). Fluorescence images were obtained using an upright Axioscope microscope equipped with a Zeiss CCD camera (16 bits; 1280 × 1024 ppi) using Axiovision software (Zeiss) and ImageJ 1.51j.

### Animals

Three and fifteen months-old male mice, *Mus musculus*, strain C57BL/6 and APP23 (maintained on the same background) were used. The animals were housed in plastic cages with stainless steel mesh lids under the standard laboratory condition with temperature 22–24°C, food and water available *ad libitum*, humidity 55% ±10% and 12/12 h light/dark cycle (lights on at 7 AM).

### Gene Expression Analysis

RNA was extracted using the Qiagen RNeasy lipid tissue kit according to the manufacturer’s instructions. RNA concentrations were measured using a NanoDrop 2000 (Thermo Fisher Scientific). First strand synthesis and quantitative real-time-PCR amplification were performed in a one-step, single-tube format using the Rotor-Gene SYBR^®^ Green RT-PCR kit from Qiagen according to the manufacturer’s protocol in a total volume of 20 μl and gene specific QuantiTect Primer Assays (Qiagen). Thermal cycling and fluorescent detection were performed using the Rotor-Gene Q real-time PCR machine (model 2-Plex HRM) (Qiagen). Resulting data were analyzed using the HMBS gene as an internal standard to normalize transcript levels. All quantitative real-time PCR reactions were run in technical triplicates and mean cycle threshold (ct) -values for each reaction were taken into account for calculations. Ct values were calculated by the Rotor-Gene Q Software (version 2.0.2) and transformed into virtual mRNA levels according to the formula: virtual mRNA level = 10 × [ct(target) − ct(standard)]/slope of standard curve.

### Histochemistry

Brain sections (14 μm thickness) were prepared from fresh snap-frozen brains using a cryostat (Leica CM 3050S) with the knife set at -23°C. Three sections of the brain of the same animal were collected on one microscope slide. For staining, the slices were thawed for 20 min at RT, fixed with PFA/4% Sucrose for 30 min at RT. After washing 10 min with 1× PBS, incubation with Triton 0.2% in 1× PBS for 2 h at RT was followed by incubation with Triton 0.05% for 10 min at RT. The slides were covered with Blocking Solution (BS)(10% FCS in 1× PBS) for 2 h at RT. The primary antibody was diluted in BS and applied over-night at 4°C. Subsequently, incubation for 3× 10 min with Triton 0.05% at RT was followed by incubation with the secondary antibody coupled to Alexa488 or Alexa568, diluted 1:500 in BS, at 37°C for 1.5 h. After a 3× 15 min washing-step with Triton 0.05% and 1× 5 min with 1× PBS, cell nuclei were counterstained with DAPI and after the last washing step with 1× PBS for 5 min, cover slips were mounted using VectaMount. Zinpyr-1 staining was performed at a final concentration of 10 μM and incubation time of 1 h at RT. Subsequently, the sections were counterstained with DAPI. Images were taken with a Zeiss LSM710 confocal microscope. For image analysis, center and border zone of plaques were determined using ImageJ. Zinpyr1 signal intensity was measured of 50 plaques using the “plot profile” function along the diameter of a plaque crossing the center. A clear difference between center and border zone in Zinpyr1 fluorescence intensity can be observed with the border zone displaying a mean value of 60% of fluorescence intensity of the center zone with a steep drop between center and border zone. Using this information, images were thresholded and two masks were created from Zinpyr1 fluorescence selecting areas above (center zone) and below 60% (border zone) of average fluorescence intensity of Zinpyr1 of the center of a plaque. These masks were used to create a selection to measure S100 signal intensity inside the respective zones of a plaque.

#### Protein Fractionation

To obtain P2 fractions from brain regions, the regions were dissected, and 1 g tissue was homogenized in 10 ml Buffer A containing protease inhibitor mixture (complete mini EDTA free, Roche). Cell debris and nuclei were removed by centrifugation at 3,200 rpm for 15 min resulting in supernatant S1 (soluble fraction) and pellet P1 (membrane associated fraction). Supernatants (S1) were centrifuged for 20 min at 11,400 rpm, resulting in S2 (soluble fraction) and P2 (crude synaptosomal fraction). The resulting pellet P2 was resuspended in homogenization buffer to measure protein concentration by Bradford analysis followed by western blotting.

#### Western Blotting

Proteins were separated by SDS-PAGE and blotted onto Nitrocellulose membranes. Immunoreactivity was visualized using HRP-conjugated secondary antibodies and the SuperSignal detection system (Pierce, Upland, United States). Evaluation of bands from Western blots was performed using ImageJ v1.52c. Three independent experiments were performed, and blots imaged using a MicroChemi Imaging System from Biostep. The individual bands were selected, and the integrated density was measured. All WB bands were normalized to β-III-Tubulin and the ratios averaged and tested for significance.

### Statistics

Statistical analysis was performed using Microsoft Excel and averages tested for significance using SPSS version 20. For comparisons, analysis of variance (ANOVA) was performed followed by *post hoc* tests for within group comparisons. Data are shown as mean ± SEM. Significance levels were set at *p* < 0.05 (<0.05^*^; <0.01^∗∗^; <0.001^∗∗∗^).

## Results and Discussion

### S100A6 and S100B Show Brain Region and Cell Type Specific Expression

To elucidate the origin of S100 proteins in brain parenchyma, in a first set of experiments, we analyzed expression of S100A6, S100A8, and S100B *in vitro* using 14 days old rat hippocampal neuronal cultures. For S100A6 and S100B, we detected immunoreactive signals mostly in astrocytes, but also a weak signal in neurons (Supplementary Figure S1A). S100A8 immunoreactive signals were found in neurons and glial cells (Supplementary Figures S1B,C) revealed by co-staining with a marker for astrocytes (Glial fibrillary acidic protein, GFAP) and a marker for neurons (Microtubule-associated protein 2, MAP2).

**FIGURE 1:**
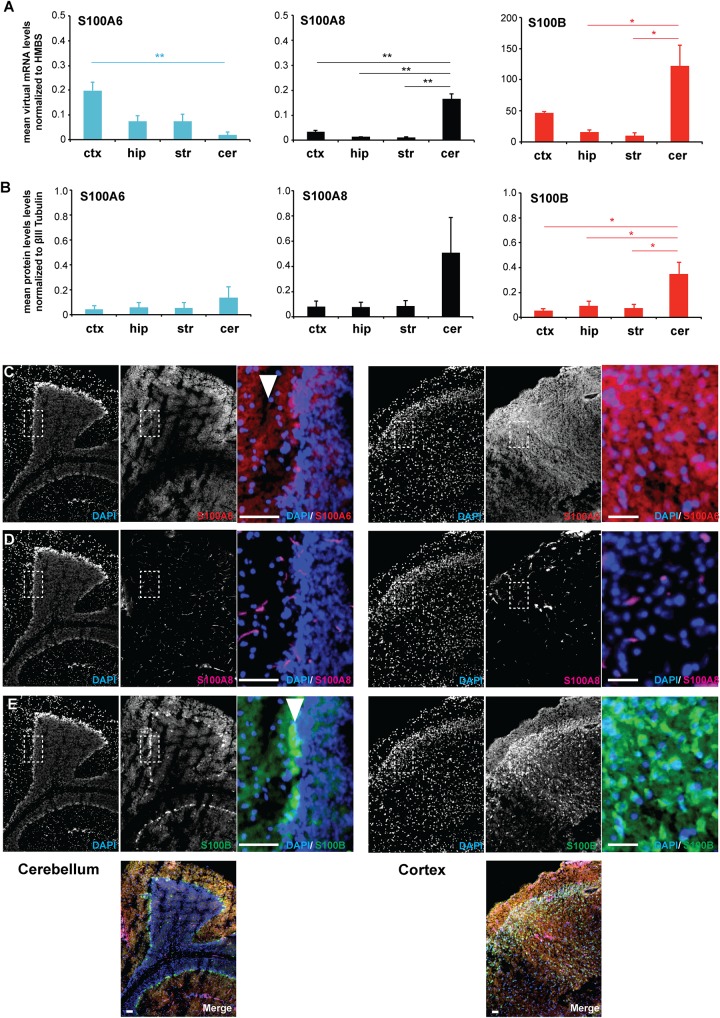
S100A6 and S100B show brain region and cell type specific expression *in vivo*. **(A)** RNA lysate was obtained from the cortex (ctx), hippocampus (hip), striatum (str), and cerebellum (cer) of WT mice. Expression analysis for S100A6, S100A8, and S100B shows that the genes are expressed in all brain regions with S100A6 showing the highest expression in the cortex, S100A8 in the cerebellum, and S100B in the cortex and cerebellum (*n* = 3) [one-way ANOVA, *F*_S100A6_ = 8.319, *p*_S100A6_ = 0.008; *F*_S100A8_ = 40.602, *p*_S100A8_ = < 0.0001; *F*_S100B_ = 9.043, *p*_S100B_ = 0.006; Bonferroni *post hoc* analysis: S100A6: a significant difference was detected between ctx and cer (*p* = 0.008). S100A8: a significant difference was detected between ctx and cer (*p* = 0.000254), hip and cer (*p* = 0.000857), and str and cer (*p* = 0.000818). S100B: a significant difference was detected between hip and cer (*p* = 0.014), and str and cer (*p* = 0.01)]. **(B)** The soluble protein fraction (S2) from protein lysate was obtained from the cortex (ctx), hippocampus (hip), striatum (str), and cerebellum (cer) of WT mice (*n* = 3). No regional differences were detected on the protein level for S100A6 and S100A8 (one-way ANOVA, *F*_S100A6_ = 0.576, *p*_S100A6_ = 0.647; *F*_S100A8_ = 2.209, *p*_S100A8_ = 0.165). A significant difference was detected for S100B (one-way ANOVA, *F*_S100B_ = 7.118, *p*_S100B_ = 0.012). Bonferroni *post hoc* analysis reveals a significant higher level of S100B in cer compared to ctx (*p* = 0.0228), cer compared to hip (*p* = 0.04934), and cer vs. str (*p* = 0.03557). **(C)** Immunohistochemistry performed on WT brain sections shows S100A6 protein expression in the cortex and cerebellum. In the cerebellum, S100A6 shows high expression in the molecular layer (arrow). **(D)** S100A8 expression is absent in neurons and glial in both cerebellar and cortical regions. S100A8 signals originate from blood vessels. **(E)** S100B is expressed in cortex and cerebellum. In the cerebellum, S100B is highly expressed specifically in the Purkinje cells (arrow). **(C–E)** Scale bars = 50 μm.

In order to investigate whether the results obtained *in vitro* are representative for the *in vivo* situation, in the following set of experiments we analyzed RNA lysates from different brain regions (cortex, hippocampus, striatum, cerebellum) obtained from adult wildtype (WT) C57BL/6 mice. mRNA expression analysis for S100A6, S100A8, and S100B shows that the genes are expressed in all brain regions. However, S100A6 shows the highest expression in the cortex, and S100A8 in the cerebellum, and S100B in the cortex and cerebellum (Figure 1A). mRNA expression levels were confirmed on the protein level for S100B, with the highest expression in the cerebellum (Figure 1B).

Based on this expression pattern, we selected the cortex and cerebellum for subsequent immunohistochemical analysis to identify protein expression and localization of S100A6, S100A8, and S100B in these brain regions. S100A6 protein expression was found in both brain regions (Figure 1C). In the cerebellum, S100A6 seems to be enriched in the molecular layer. In contrast, S100B is very specifically expressed in the Purkinje cells of the cerebellum. Lower expression of S100B was detected in other regions of the cerebellum and in the cortex (Figure 1E). The expression of cerebellar S100B is well established in the developing mouse cerebellum where it is proposed to be involved in interactions with vimentin, to participate in neurite extensions and to have neurotrophic activities (Hachem et al., 2007). However, S100B-deficient mice exhibit normal cerebellar development (Bluhm et al., 2015). On the other hand, systematically elevated S100B levels during neurodevelopment impair cerebellar oligodendrogenesis and myelination (Santos et al., 2018). In the cortex, S100B protein expression is the highest in layer IV. S100A8 protein expression in turn seems nearly absent in neurons and glial in both cerebellar and cortical regions. S100A8 signals almost exclusively originate from blood vessels, either from endothelial cells forming the wall of blood vessels or from protein in blood (Figure 1D). Thus, the expression of S100A8 is different in dissociated cell culture from the *in vivo* situation. Alternatively, altered expression may be due to differences between rat used for cell culture, and mouse tissue used for *in vivo* studies.

### Altered Localization of S100 Proteins in the Brain of APP23 Mice

**FIGURE 2:**
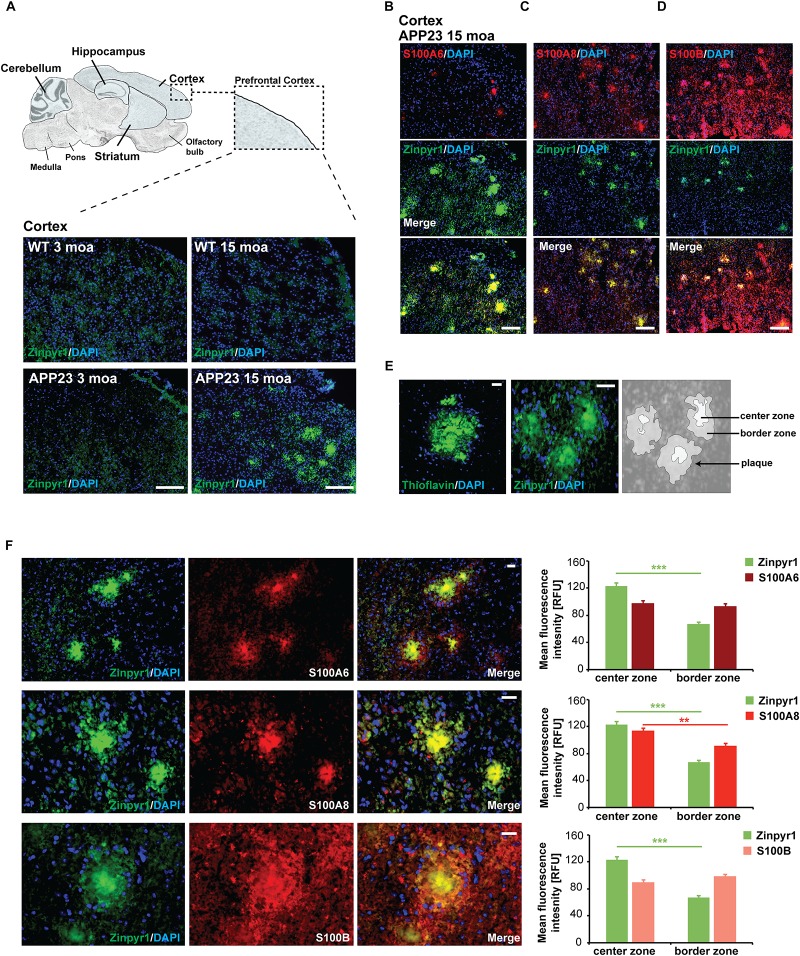
Altered localization of S100 proteins in the brain of AP23 mice. **(A)** APP23 mice were compared to controls at 3 moa and 15 moa. Representative images of mouse prefrontal cortex are shown. A severe plaque-pathology was observed in 15 moa APP23 mice in this brain region. Plaques were visualized by Zinpyr-1 staining and cell nuclei labeled using DAPI. **(B)** In 15 mao APP23 mice, S100A6 signals co-localize with Aβ plaques together with **(C)** S100A8, and **(D)** S100B. Signal intensities have been adjusted to S100 fluorescence co-localizing with plaques leading to very low fluorescent signals in the surrounding tissue. **(E,F)** Visualization of plaques using Thioflavin or Zinpyr1 reveals strong fluorescence in the center of plaques and weaker fluorescence in the border zone corresponding to the aggregation state of Aβ. **(F)** S100A8 is significantly higher enriched in the center of Aβ aggregates (20–25 plaques each from *n* = 3 mice, *t* test, *p* = 0.0077). S100A6 is found in the center and periphery of Aβ plaques (*p*_S100A6_ = 0.3896). In contrast, S100B is found in the center zone and is slightly enriched in the border zone (*p*_S100B_ = 0.1217). **(A–D)** scale bars = 300 μm, **(E,F)** = 50 μm.

Given that a role of S100 proteins has been proposed in AD, we next analyzed whether the expression and distribution of S100A6, S100A8, and S100B is altered in APP23 mice compared to controls before the onset of amyloid plaque pathology (3 months of age, moa) and at old age with full amyloid plaque pathology (15 moa). Pathology was confirmed by visualization of plaques in brain sections of mice. Plaques were found in the hippocampus and cortex of 15 months old APP23 mice, but in line with the literature (Maier et al., 2015), no plaques were detected in the cerebellum. The prefrontal cortex of mice was then analyzed (Figure 2A).

In APP23 mice cortex (15 moa, plaque positive), S100A8 immunoreactive signals maintain their association with blood vessels. However, in addition, S100A8 signal co-localizes with Aβ plaques (Figure 2B). Similarly, S100A6 and S100B localize to Aβ plaques (Figures 2C,D). Several methods exist to visualize the plaques. Among them staining with Thioflavin is commonly used. Thioflavin detects the presence of amyloid fibrils (Xue et al., 2017). Cross-linked fibrils form the center of amyloid plaques and increasing aggregation has been hypothesized to originate from fibrils that radiate out from a center recruiting Aβ monomers and oligomers (Drolle et al., 2014) that are found in the periphery of a plaque. Therefore, staining with Thioflavin results in a strong signal in the center of plaques and weaker signals from the border zone. Like Thioflavin, Zinpyr-1, a fluorophore labeling zinc associated with Aβ aggregates results in a strong labeling of plaques. As zinc ion levels are elevated in amyloid plaques (Stewart and Radford, 2017), and in particular in β-sheets associated with fibrillar assemblies (Rambaran and Serpell, 2008), also zinc staining shows higher fluorescence in the center of plaques (Figures 2E,F). Analysis of the distribution of S100 proteins within the center and border zone of plaques revealed that S100A8 is significantly enriched in the center of Aβ plaques. In contrast, S100B localizes to the center, but more so in the border zone of plaques (Figure 2F). S100A6 is found both in the center and border zone of plaques (Figure 2F).

### Altered Concentrations of S100A8 Protein in the Brain of APP23 Mice

**FIGURE 3:**
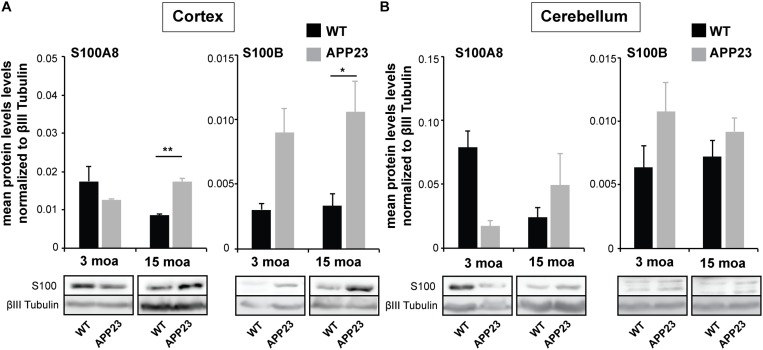
Altered concentrations of S100A8 proteins in the brain of APP23 mice. Brain lysate was obtained from the cortex and cerebellum of control and APP23 mice at 3 and 15 moa. P2 fractions were used for analysis (*n* = 3). **(A)** Left, In Cortex, a statistically significant difference was found between APP and WT mice at 15 moa, but not a 3 moa. S100A8 levels were significantly higher in APP23 mice at 15 moa compared to WT mice (two-way ANOVA followed by Bonferroni *post hoc* test, *p* = 0.0091). Right, S100B levels were higher in APP23 mice at each time-point with a significant difference between WT and APP23 mice at 15 moa (two-way ANOVA followed by Bonferroni *post hoc* test, *p* = 0.0473). **(B)** Left, In Cerebellum, protein levels of S100A8 decrease during aging in the cortex of WT mice but not in APP23 mice. However, two-way ANOVA analysis does not reveal significant differences. Right, Similarly, no significant time or genotype dependent differences were observed for S100B in the cerebellum (two-way ANOVA).

Given that changes in the expression of S100 family members have been reported during aging in humans and WT mice (Swindell et al., 2013), we next investigated if alterations on protein level also occur for S100A8 and S100B, and whether the differences found in WT animals are also observed in APP23 mice. To that end, brain lysates were prepared and sub-fractionated. To exclude the influence from blood vessels, P2 (synaptosomal fractions) were used for the analysis. The results show that aged WT mice have decreased protein levels of S100A8 in the P2 fraction both from the cortex and cerebellum (Figures 3A,B). However, this is not the case in the APP23 mice. A significantly higher level of S100A8 was found in the in the P2 fraction from the cortex of APP23 mice compared to WT at 15 moa (Figure 3A). The increased S100A8 levels in the cortex of APP23 mice after development of AD pathology are in line with the previous observed shift from S100A8 signals mainly associated with blood vessels to the center of Aβ plaques. S100B levels, on average, are higher in APP23 mice compared to controls in both brain regions as early as 3 moa, with a significant higher S100B level in the cortex of APP23 mice at 15 mao (Figure 3A).

### Influence of S100 Proteins on Aβ Aggregation

**FIGURE 4:**
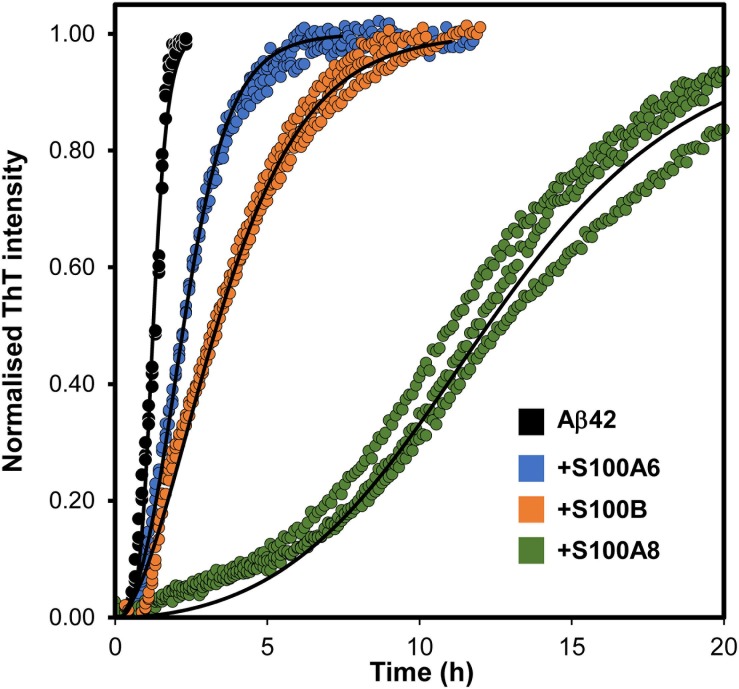
Effect of S100 proteins on Aβ42 aggregation. Fibril formation of monomeric 5 μM Aβ42 (black, *t*_1/2_ = 0.8 h) in 50 mM Hepes (pH 7.4) with 1.1 mM CaCl_2_ at 37°C under quiescent conditions in the presence of S100A6 (blue, *t*_1/2_ = 1.3 h), S100B (orange *t*_1/2_ = 2.2 h) and S100A8 (green, *t*_1/2_ = 3.5 h), all at 15 μM. Plots represent averaged normalized intensity curves, fitted to a secondary nucleation model, obtained from three independent replicates for each of the tested conditions. *t*_1/2,_ reaction half times.

Considering the recent finding that S100B is a calcium-tuned suppressor of Aβ42 aggregation (Cristóvão et al., 2018), and to gain further mechanistic insights into the yet unclear roles of S100 proteins near amyloid plaques, we next investigated if other S100 proteins might play similar regulatory effects. For that, we have conducted ThT monitored *in vitro* kinetic experiments of amyloid formation, in which we tested the potential suppressing effects of S100A6 and S100A8 over the aggregation of monomeric Aβ42. Similar to what has been reported for S100B (Cristóvão et al., 2018), the calcium-bound forms of S100A8 and S100A6 also delay Aβ42 aggregation (Figure 4). Indeed, the increased half-times (*t*_1/2_) of the Aβ42 aggregation determined in the presence of the analyzed S100 proteins reveal that S100A8 has the strongest effect (∼7.5× increase), under the tested conditions. For S100B this effect has been shown to result from a direct physical interaction with Aβ42 (Cristóvão et al., 2018) and at this stage we can only speculate that similar phenomena might also be occurring for S100A6 and S100A8. In support of this possibility is the evidence that the S100A8-containing heterodimer calprotectin interacts with Aβ40, also influencing its aggregation (Lee et al., 2018).

## Conclusion

S100B, S100A8, and S100A6 are among the most prominent brain expressed S100 proteins and are all upregulated upon traumatic brain injury, aging, and neuronal damage. Understanding the physiology of S100 proteins in the brain through a systematic assessment of their relative distribution and expression levels in healthy, aged and diseased states is a critical first step to establish the molecular mechanisms through which these important signaling molecules act on AD neurodegeneration. Neuroinflammation is a well-established hallmark in AD, and glia-neuronal interactions mediated through the chronic release of glia-derived cytokines including Interleukin-1 and S100B are postulated to be key for the neurodegeneration process (Griffin et al., 1998). It is also emerging that early inflammation stages, prior to plaque formation, encompass a number of released glial mediators with the potential to regulate AD processes (Cuello, 2017). It is our contention that S100 proteins may play important roles in the early AD stages and we have therefore in this work interrogated about their location and distribution in the brain, focusing on S100A8, S100A6, and S100B. Recent work from our laboratories has shown that S100B has indeed novel chaperone-like functions capable to mitigate the earliest stages of Aβ amyloid aggregation and its toxicity (Cristóvão et al., 2018) and to buffer neurotoxic zinc through its active chelation that impacts on trace metal homeostasis (Hagmeyer et al., 2017). These findings suggest that prior to pro-inflammatory and disease-aggravating roles in later disease stages, S100 proteins may engage in new protective activities related to amyloid aggregation processes, which might be amenable to future pharmacological intervention to mitigate AD progression.

## Ethics Statement

For primary neutrons from rat: Animal experiments were performed in compliance with the guidelines for the welfare of experimental animals issued by the Federal Government of Germany and approved by the Regierungspräsidium Tübingen and the local ethics committee at Ulm University (Ulm University, ID: O.103). For APP23 mice: Animal experiments were performed in compliance with the guidelines for the welfare of experimental animals issued by the Federal Government and approved by the local ethics committee of the University of Modena and Reggio Emilia.

## Author Contributions

CG and AG conceived the study and wrote the manuscript. SH, MR, and JSC conducted the experiments. AV and MZ generated the APP23 mice. All authors edited and approved the manuscript.

## Conflict of Interest Statement

The authors declare that the research was conducted in the absence of any commercial or financial relationships that could be construed as a potential conflict of interest.
