# Supplementary material for: Distribution and Relative Abundance of S100 Proteins in the Brain of the APP23 Alzheimer’s Disease Model Mice
Source: Front Neurosci. 2019 Jun 20;13:640. doi: 10.3389/fnins.2019.00640 (PMC6596341; doi:10.3389/fnins.2019.00640)
Supplement: Supplementary file 1 [file Image_1.pdf]

Distribution and relative abundance of S100 proteins in the brain of the APP23 Alzheimer's Disease model mice

Simone Hagmeyer, Mariana A. Romão, Joana S. Cristóvã, Antonietta Vilella, Michele Zoli, Cláudio M. Gomes, Andreas M. Grabrucker

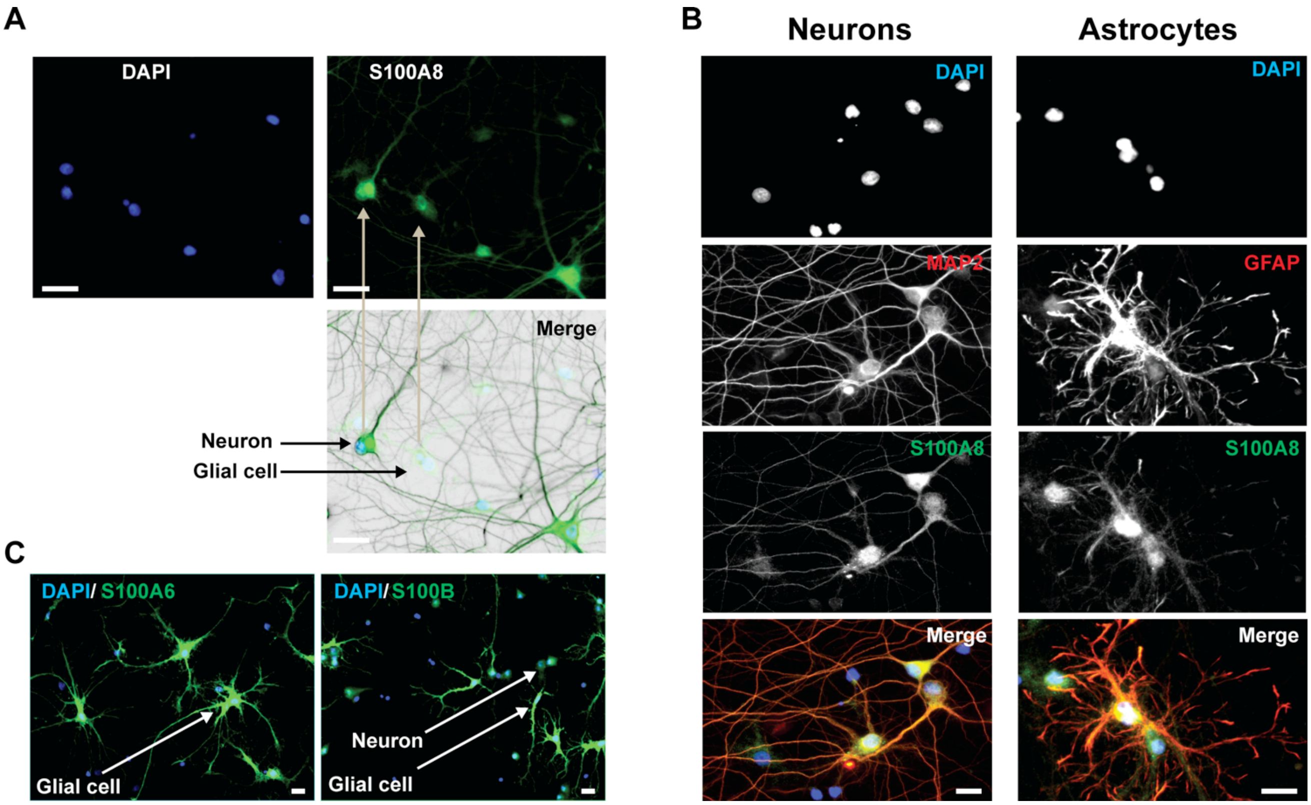

Figure S1 - S100A6, S100A8, and S100B are expressed in glial cells and neurons *in vitro*. Rat hippocampal neuronal cultures were prepared and immunocytochemistry performed at DIV 14. **A)** S100A8 immunoreactive signals (green) are found in neurons and glial cells. Nuclei are visualized by DAPI (blue). Merged image shows dark staining of neurons by MAP2. **B)** S100A8 co-localizes with markers for astrocytes (GFAP) and for neurons (MAP2). **C)** Immunoreactive signals for S100A6 and S100B were detected in astrocytes and residually in neurons. **(A-C)** Scale bars = 50 μm.
